# On Some of the Relations of Volition to the Physiology and Pathology of the Spinal Cord

**Published:** 1848-05

**Authors:** 


MEDICAL SOCIETY OF LONDON, FEB. 14, 1848.

Mr. W. F. Barlow read a part of a paper—

*On some of the Relations of Volition to the Physiology and Pathology of the Spinal Cord.*

He enforced the necessity, not only of studying the divisions of the nervous system, but of contemplating it as a whole when all its functions blended with and modified each other, and drew a contrast between the merely dynamic life of the headless, as compared with the existence of the perfect being. The power of the will in preventing certain reflex movements, its nice and varied adaptation to the opposite characters of the movements it modified, its influence over centric as well as eccentric motions, over those springing from emotions as well as over those having a purely physical source, its degree of energy, as affected by age, sex, temperature, &c., were severally touched upon. Respiration, as a mixed function, was considered; no function was at once so obviously independent of, and yet so strictly obedient to, the will. Its nature as a merely vital function, and that more complex and perfect state of it, in which it was made subservient to the highest intellectual purposes, as common speech and oratory daily exemplified, were respectively dwelt upon. The different effects of various stimuli in exciting reflex respiratory movements, according as the will interfered or not; the marked effect of temperature in producing them, as well as the relation of the power of temperature to the extent of the surface impressed, (a matter to be regarded in the treatment of disease,) were pointed out. Volition, as an auxiliary in diseases of the respiratory organs, and the dangers of its withdrawal in peculiar states of them; its state in very early life in respect of the convulsions which are then proverbially common; various instances in which feebleness of the will and liability to spasm exist together; the connection between the will and the motor force of the "true" spinal marrow, which supplies the material through which it is manifested; the power of the will compared with that of the involuntary actions wherewith it occasionally contended; the resistance successfully opposed by it in certain cases, to the action of galvanism and strychnine; the various states of volition in paralysis, and the consequent varieties presented by the disease, were amongst the chief matters presented by the author. The writings of Dr. Marshall Hall, Sir Charles Bell, Legallois, Flourens, Professor Muller, Mr. Lawrence, Dr. William Budd, and the Physiological notes of Dr. Baly, were severally referred to; and some interesting remarks of John Hunter, on the relation of volition to spasmodic affections, were read to the Society.

FEBRUARY 21, 1848.

Mr. Barlow concluded his paper, and drew attention to some phenomena of paralysis, certain of the worst forms of idiocy, and some of the symptoms of hydrophobia, in further illustration of the subject.

Mr. Bishop in reference to the influence of volition over the vocal organs, remarked, that cutting the tonsils, uvula, and other operations on the throat, in these cases only did temporary good in those instances in which the patient had previously a certain degree of power over the emotions. No operation was of service in case of confirmed stammer.

Mr. Clarke had seen no case in which operation, on the throat, for stammering, had been of any permanent benefit.

Mr. Pilcher said, that the effect of operation in these cases was similar to that of any other excitement; there might be a temporary relief but no permanent effect.

Mr. Bishop thought, with reference to the removal of the uvula, that this organ had some influence on the articulation of gutturals, and its removal would so far interfere with the voice in singing.

Dr. Willshire inquired whether any of the members had noticed, in those cases in which the uvula had been removed, that there had been disease of the follicles at the base of the tongue? To moisten the base of the tongue, and its adjacent parts, seemed to be the function of the uvula. He related a case in which intense agony, lasting for some days, followed the excision of the uvula.

Mr. Pilcher thought there was no great harm in snipping off a portion of the tonsils. We were unacquainted with the function of the uvula. He related a case in which, after a removal of this body, a lady lost a beautiful high note of the voice, which never returned.

Mr. Hancock did not call proceedings such as those referred to, surgical operations; they were not worthy of that name. The tonsils might sometimes require excision, for the relief of obstruction in the throat. He recollected a case in which alarming hæmorrhage followed the excision of a superficial portion of the tonsil. There was no hæmorrhagic tendency in the patient, who was a boy.

Mr. Bishop remarked that the case detailed by Mr. Pilcher illustrated the value of every organ of the vocal apparatus. He had never seen hæmorrhage result from the removal of the tonsils.

Some conversation followed, chiefly in condemnation of the cruelty and uselessness of mutilating the throat in cases of stammering.

Dr. Willshire exhibited specimens of spleen, mesenteric glands, lungs and bronchial glands, all in a state of extreme tuberculization, and the rectum and a portion of the colon presenting the characters of certain forms of entero-colitis met with in infantile life—viz., hypertrophy and ulceration of the mucuous follicles, with inflammation of the edges of the longitudinal plicæ of the intestinal lining membrane. These morbid specimens were taken from the body of a child, aged one year and eleven months, who had died of scrofulous meningitis. On examination of the brain, the vessels of the meninges were found greatly congested; some albuminous deposite along the course of the larger ones, over the hemispheres, with here and there a little semi-concrete yellow exudation. About five ounces of fluid were observed in the ventricles, and the central portions of the brain were much softened. At the base of the brain there was much semi-concrete yellow exudation and hyaline matter deposited. Dr. Willshire observed that this was a remarkable exception to what we usually see in cases of the so-called acute hydrocephalus, inasmuch as we almost constantly observe the yellow and grey effusions assume a granular or spheroidal, or, in other words tubercular, development. It is true that here and there a few granules were seen, but the granular development did not characterize the case. Although they were wanting, Dr. Willshire still could not help regarding the case as one of scrofulous inflammation, and the yellow concrete matter as of scrofulous origin. Dr. Willshire alluded to the views held on the Continent, in opposition to those very prevalent amongst ourselves, relative to acute hydrocephalus.—*London Lancet.*

